# Anti-Inflammatory Dimethylfumarate: A Potential New Therapy for Asthma?

**DOI:** 10.1155/2013/875403

**Published:** 2013-03-27

**Authors:** Petra Seidel, Michael Roth

**Affiliations:** ^1^Pulmonary Cell Research, Department of Biomedicine, University of Basel, Hebelstraße 20, 4031 Basel, Switzerland; ^2^Pneumology, Department of Internal Medicine, University Hospital Basel, Petersgraben 4, 4031 Basel, Switzerland

## Abstract

Asthma is a chronic inflammatory disease of the airways, which results from the deregulated interaction of inflammatory cells and tissue forming cells. Beside the derangement of the epithelial cell layer, the most prominent tissue pathology of the asthmatic lung is the hypertrophy and hyperplasia of the airway smooth muscle cell (ASMC) bundles, which actively contributes to airway inflammation and remodeling. ASMCs of asthma patients secrete proinflammatory chemokines CXCL10, CCL11, and RANTES which attract immune cells into the airways and may thereby initiate inflammation. None of the available asthma drugs cures the disease—only symptoms are controlled. Dimethylfumarate (DMF) is used as an anti-inflammatory drug in psoriasis and showed promising results in phase III clinical studies in multiple sclerosis patients. In regard to asthma therapy, DMF has been anecdotally reported to reduce asthma symptoms in patients with psoriasis and asthma. Here we discuss the potential use of DMF as a novel therapy in asthma on the basis of *in vitro* studies of its inhibitory effect on ASMC proliferation and cytokine secretion in ASMCs.

## 1. Introduction

Asthma is a disease of the airways characterized by chronic inflammation associated with airway hyperresponsiveness (AHR) and airway wall remodeling. In the past decades, numerous immunological studies of lung fluids and animal studies suggested that asthma is a disease caused by the deregulation of the immune response to inhaled or eaten allergens that leads to structural changes of the airway tissue which increase with the duration of the disease [1–3]. New clinical studies, especially in childhood asthma, suggest that inflammation and remodeling occur independent of each other in parallel or even that airway wall remodeling especially of the airway smooth muscle occurs before any signs of inflammation can be found [4–7]. Therefore the question if the pathophysiology of the airway smooth muscle cell is crucial for the pathogenesis of asthma was reactivated [8].

**Figure 1:**
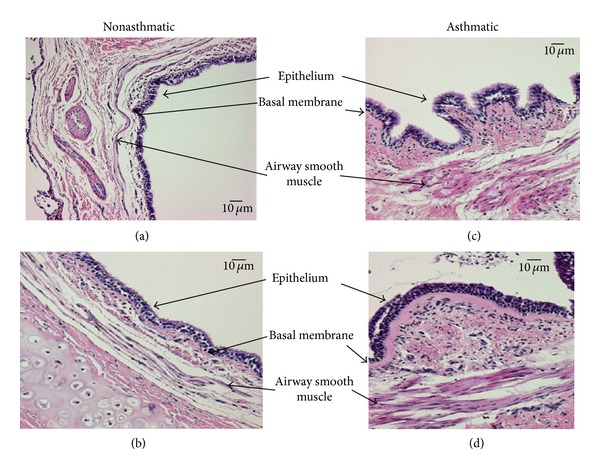
Histological tissue sections of two patients with moderate asthma and two nonasthma controls. Arrows indicate the loss of epithelium integrity, the increase of the basement membrane thickness, and the increased number of ASM bundles in the asthmatic airways (c, d), compared to nonasthmatic airways (a, b).

The increased mass of cells within airway smooth muscle (ASM) bundles is one of the most striking pathological features in the asthmatic airway and inversely correlates with lung function in asthma [9]. The role of the airway epithelium as a master regulator of airway wall forming cells has recently been demonstrated; however, the mechanism(s) by which a deranged epithelium affects the underlying cell types has to be studied in more detail [10]. In Figure 1, we provide two examples of the airway wall obtained from nonasthmatic adults and from two moderate asthmatics. Both tissue sections of the asthmatic airways demonstrate the well-known loss of epithelium integrity, the significant increase of the basement membrane thickness, and the increased number of ASM bundles. In contrast, there is no clear increase of the thickness and structure of the subepithelial fibroblast/myofibroblast cell layer (Figure 1).

Recent studies support the hypothesis that the increase of the ASM bundles in the airway wall of asthma patients is an early event developing independently in parallel to inflammation [4–7]. Comparing the airway wall structure in biopsy material of 53 school children with treatment-resistant asthma to that of 16 healthy age-matched controls provided evidence that remodeling, especially the increase of ASM bundle size, was independent of proinflammatory Th2-cell derived cytokines (IL-4, IL-5, and IL-13), while eosinophil counts varied over a wide range [4]. Assessing endobronchial biopsy specimen of ASM obtained from 47 wheezing preschool children and 21 nonwheezing controls, it was documented that an increased mass of ASM occurred in the majority of wheezing children [7]. In a nonhuman primate model of asthma and COPD, a striking rearrangement of the smooth muscle cell bundles from a nonstructured into a spiral-like formation surrounding the airway was described [11, 12]. These findings suggested that allergic as well as nonallergic asthma triggers induce a pathological reorganization of ASM bundles by an unknown mechanism. Furthermore, it was reported that inhalation of either methacholine or house dust mite allergens by volunteering patients with mild asthma leads to airway wall remodeling within only eight days, which was prevented by inhalation of a long-acting *β*2-agonist [5]. In addition, removal of ASM cells by thermoplasty significantly reduced asthma symptom of several years and is today regarded as a therapeutic option for severe asthma [13–15]. Several *in vitro* and *in vivo* studies have shown that ASM cells (ASMCs) secrete a variety of mediators, which enable them to interact with immune cells and to modulate the inflammatory response and remodeling in asthma [9, 16, 17]. Together these observations strongly support the central role of ASMC in the pathogenesis of asthma and therefore they are interesting targets for asthma therapy [18, 19].

Inhaled long-acting *β*2-agonists (LABAs) combined with glucocorticoids (GCs) remain the most effective therapy for asthma. However, a considerable number of asthma patients do not respond to inhaled GCs [20]. In addition, the current therapy only controls disease symptoms, but none of the existing asthma medications cures the disease. This emphasizes a need for new therapeutic options to treat asthma more efficiently [21–23]. 

Dimethylfumarate (DMF) is a potent anti-inflammatory medication for psoriasis and it has also been shown to suppress inflammation in other chronic inflammatory diseases, especially MS [24]. Interestingly, DMF has been anecdotally reported to reduce asthma symptoms in patients suffering from asthma and psoriasis. In experimental studies, DMF inhibited proliferation and proinflammatory transcription factors as well as the secretion of asthma-relevant cytokines in primary human lung cells [25–28]. In this paper we describe how ASMC-derived CXCL10, CCL11, or RANTES may contribute to airway inflammation in asthma and how these chemokines can be controlled by the anti-inflammatory action of DMF.

## 2. Airway-Smooth-Muscle-Cell-Derived Chemokines Contribute to Airway Inflammation

ASMC hyperplasia and hypertrophy in asthmatic airways had already been described in 1922 and was considered as the main cause of AHR [8]. Interestingly, in recent years it became evident that ASMCs hypertrophy may precede inflammation and that ASMC are an important source of inflammatory mediators and therefore actively contribute to airway inflammation [4–6]. Proinflammatory cytokines activate the ASMC to secrete further chemokines that subsequently attract immune cells such as mast cells [16] or T lymphocytes [9] into the ASM bundle. These immune cells then interact with ASMC and alter their contractile function, enhance proliferation, and further amplify the secretion of proinflammatory factors. For instance, it has been reported that mast-cell-derived tryptase enhanced ASMC contractility [29], induced ASMC proliferation [30], and increased TGF-*β*1 secretion [31]. Similarly, T lymphocytes infiltrated the asthmatic ASM bundle and induced ASMC proliferation [9, 32]. In the following, we will focus on ASMC-derived chemokines CXCL10, CCL11 (eotaxin), and RANTES, which are crucially involved in the trafficking of immune cells into the airway in asthma.

## 3. CXCL10 (IP-10)

ASMC-derived CXCL10 is a potent chemoattractant for human lung mast cells [33]. In a disease-specific pattern, ASM bundles are infiltrated by activated mast cells in asthma, as this pathology was neither observed in patients with eosinophilic bronchitis nor in nonasthmatic controls [16, 34]. Consequently, it was hypothesized that the ASMC itself attracts mast cells by secreting chemokines such as CXCL10. This assumption was supported by studies in ASMC of asthma patients, which expressed higher levels of CXCL10 than ASMC derived from nonasthmatic controls. In addition, CXCL10 has been detected in the ASM bundles of asthmatic airway biopsies only and all mast cells within the ASM bundles expressed CXCR3, which is the receptor for CXCL10 [33]. A wide range of asthma relevant stimuli have been reported to increase signaling pathways that may lead to incerased CXCL10 secretion [35–38].

CXCL10 is secreted by ASMC after stimulation with proinflammatory cytokines such as TNF-*α*, IFN-*γ*, or IL-1*β*, which activated MAPK JNK, NF-*κ*B, STAT 1, and the transcriptional coactivator CREB-binding protein [25, 39–41]. In addition, CXCL10 secretion by ASMC is sensitive to changes in cellular glutathione (GSH) levels [27], suggesting a link of this signaling pathway to the asthma-associated upregulation of mitochondria, which control the cellular redox system [42]. Interestingly, the thiazolidinedione ciglitazone strongly inhibited cytokine-induced CXCL10 protein without affecting CXCL10 mRNA level, suggesting that CXCL10 is regulated on the posttranslational level in ASMC [41]. In this context, it would be of interest if the posttranscriptional regulation of CXCL10 in asthma occurs through the recently described modified translation control [43]. 

## 4. CCL11 (Eotaxin)

CCL11 is a potent eosinophil chemoattractant and eosinophilia is a prominent pathology of the asthmatic airway [44–46]. *In vivo*, the asthmatic ASM bundle showed strong signals of CCL11 immunoreactivity and CCL11 mRNA [47]. In addition, in bronchial biopsies of asthmatic patients, CCL11 expression correlated with asthma severity [44, 48]. *In vitro*, ASMC secreted CCL11 [26, 35] and it has been shown that ASMCs derived from asthmatic patients produce higher levels of CCL11 mRNA and protein than those derived from nonasthmatic controls [49, 50]. CCL11 secretion from ASMCs can be induced by Th1 and Th2 cytokines including TNF-*α*, IL-1*β*, IL-13, or IL-4 [26, 35, 51, 52] and critically involves the activation of NF-*κ*B [26, 35]. In addition to its chemoattractant function, CCL11 has been proposed to stimulate ASMC hyperplasia, as ASMCs express the CCL11 receptor CCR3, which, upon activation, induces ASMC migration but in this study did not induce ASMC proliferation [49]. However, in a different study CCL11 increased [^3^H]-thymidine incorporation and DNA synthesis and decreased the rate of apoptosis in ASMC [53].

## 5. RANTES

RANTES is a chemoattractant for eosinophils, T cells, and monocytes and thus has been linked to asthma pathology [54, 55]. In asthma, the ASM bundles are infiltrated by T lymphocytes [9] and ASMCs produce elevated levels of RANTES mRNA [56]. ASMC-derived RANTES was therefore proposed to participate in the chemotaxis of T lymphocytes to the ASM bundle. IFN-*γ* and TNF-*α* stimulated RANTES secretion from ASMC *in vitro* [26, 35, 51] and this was dependent on the activation of NF-*κ*B [26, 35], MAPK JNK [57], and AP-1 [58]. Like CCL11, RANTES participated in airway remodeling by inducing ASMC proliferation and migration [53, 59]. Interestingly, recombinant RANTES is degraded by mast cell tryptase and thereby reduced RANTES-activated chemotaxis of eosinophils [55]. Furthermore, mast-cell-derived histamin has been shown to reduce RANTES secretion by ASMC *in vitro* [60]. The inactivation of RANTES by mast-cell-derived mediators might explain the phenomenon that the asthmatic ASM bundle is infiltrated by a much higher number of mast cells than of T lymphocytes [16]. 

## 6. Dimethylfumarate (DMF)

**Figure 2:**
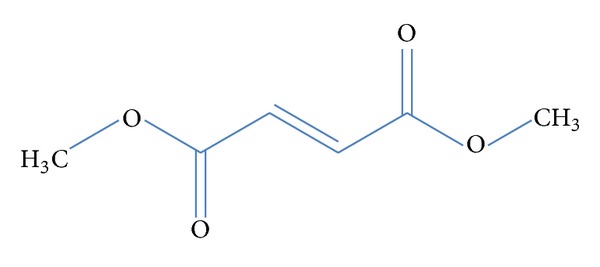
Chemical structure of dimethylfumarate.

DMF is the ester of the unsaturated dicarboxylic fumaric acid (Figure 2). The German chemist Walter Schweckendieck described the anti-inflammatory properties of DMF in 1959 [61]. Schweckendieck postulated that psoriasis is caused by a dysfunctional citric acid cycle and hypothesized that DMF is metabolized to fumaric acid, which enters the citric acid cycle and thereby inhibits the inflammatory processes. Schweckendieck tested several forms of fumarates in self-experiments and his psoriasis improved [61]. Based on his findings, the physician Günther Schäfer developed a psoriasis therapy with a mixture of fumaric acid esters. In 1989 a controlled clinical study proved the efficacy of DMF in psoriasis [62] and soon thereafter a mixture of DMF with calcium, magnesium, and zinc salts of ethyl hydrogen fumarate was registered in Germany as Fumaderm for the systemic treatment of psoriasis. Fumaderm is widely used for the treatment of moderate to severe psoriasis vulgaris in Northern Europe [24, 63–65]. In 2003 a second-generation fumaric acid derivate, BG-12, which only contains DMF in enteric-coated microtablets, has been developed (no authors listed BG 12). BG-12 has been successfully tested in phase II and III clinical studies for the oral treatment of multiples sclerosis [66–68].

## 7. Clinical Use and Pharmacokinetic of DMF

Fumaderm is administered orally in slowly increasing doses till a clinical effect is observed. The initial dose is 30 mg DMF per day, which can be increased to a maximum daily dose of 720 mg DMF [64, 65, 69]. In several clinical studies, Fumaderm showed an excellent antipsoriatic effect causing an improvement of about 75% of the baseline PASI (psoriasis area and severity index) in up to 70% of the patients tested [64, 69–71]. Even though side effects such as gastrointestinal complaints or flushing occur, Fumaderm has been shown to be very safe as long-term treatment for psoriasis with no long-term toxicity, higher risk for infections or malignancies [72].

Although Fumaderm has been used for many years, its pharmacokinetic is still poorly understood. DMF is the main ingredient of Fumaderm and is clinically most efficacious [73]. However, after oral administration of Fumaderm only, monomethylfumarate (MMF) with serum peak concentrations at around 20 *μ*M, but not DMF, was detectable in blood plasma [68, 74]. It was therefore hypothesized that DMF's hydrolysis-product MMF is the actual active compound. However, this notion has been questioned by many *in vitro* studies showing that MMF is pharmacologically less effective when compared to DMF. For instance, in human endothelial cells, DMF reduced the expression of VCAM-1, ICAM-1, and E-selectin with IC_50_ values of approximately 50 *μ*M, whereas monoethylfumarate at concentrations of 10–100 *μ*M showed no inhibitory effect [75]. Similarly, in human keratinocytes, DMF at concentrations of  7–140 *μ*M inhibited IL-1*β*-induced phosphorylation of MSK-1, whereas MMF at 140 *μ*M had no effect on MSK-1 activation [76]. DMF has been shown to rapidly react with glutathione (GSH) under physiological conditions *in vitro* [77]. It was therefore proposed that DMF is released into the bloodstream where it is absorbed by cells and conjugated to GSH, explaining why DMF is not detectable in plasma after oral intake. This assumption was supported by a study, showing that DMF-GSH-conjugate metabolites are secreted in the urine of DMF-treated psoriasis patients [78]. However, the complex pharmacokinetic of DMF makes it difficult to relate concentrations of DMF used in cell culture models to DMF concentrations in target tissue or plasma levels *in vivo*.

## 8. The Anti-Inflammatory Action of DMF in Psoriasis

Several *in vitro* and *in vivo* studies have proven the potent anti-inflammatory effects of DMF and its favorable safety profile in the treatment of psoriasis [79]. DMF reduced the proinflammatory contribution of several cell types including T lymphocytes, mononuclear blood cells, dendritic cells (DCs), endothelial cells, and keratinocytes, which are all crucially involved in the inflammatory process in psoriasis, as will be discussed in the following.

In purified human T lymphocytes, DMF inhibited the proinflammatory transcription factor NF-*κ*B and induced apoptosis [80, 81]. *In vivo* studies in psoriasis patients showed that DMF reduced the total number of peripheral blood T lymphocytes and the number of T lymphocytes in psoriatic lesions [82, 83]. Regarding its anti-inflammatory action, DMF inhibited the maturation of DC by reducing the expression of interleukin- (IL-) 12, IL-6, major histocompatibility complex (MHC) class II, cluster of differentiation (CD)80, and CD86, mainly through the inhibition of NF-*κ*B, and mitogen- and stress-activated protein kinase- (MSK-) 1. These immature DCs were shown to generate fewer IFN-*γ*- and IL-17-producing T lymphocytes [84].

In human keratinocytes and mononuclear blood cells, DMF inhibited mRNA and protein expression of CXCL8, CXCL9, and CXCL10 [85]. Furthermore, the expression of CXCL8 and IL-20 mRNA in human keratinocytes was inhibited by DMF, which was mediated by reduced MSK-1, NF-*κ*B, and cAMP response element-binding protein (CREB) activation [76, 86]. In human peripheral blood mononuclear cells, DMF reduced GSH level and upregulated heme oxygenase- (HO-) 1 expression, which resulted in the inhibition of TNF-*α*, IL-12, and IFN-*γ* secretion [87]. In addition, DMF inhibited macrophage migration inhibitory factor- (MIF-) induced human keratinocytes proliferation by reducing MSK-1, 90 kDa ribosomal S6 kinase (RSK), CREB, and JunB phosphorylation [86].

In endothelial cells, DMF inhibited the nuclear entry of NF-*κ*B, resulting in a reduced expression of TNF-induced tissue factor [88]. Furthermore, the expression of the adhesion molecules intercellular adhesion molecule- (ICAM-) 1, vascular cell adhesion molecule- (VCAM-) 1, and E-selectin on endothelial cells was inhibited by DMF, resulting in impaired lymphocyte rolling and adhesion [89]. DMF also had antiangiogenic properties by the inhibition of vascular endothelial growth factor receptor (VEGFR)2 expression on human endothelial cells [90]. 

## 9. DMF as Potential Treatment for Multiple Sclerosis

Multiple sclerosis (MS) is a chronic disease of the central nervous system, which is characterized by inflammation, demyelination, axonal loss, and glial proliferation. Currently therapy for MS is parenteral administered and is only partially effective, as patients do not remain relapse-free after treatment [91]. Clinical phase II and III studies investigating a potential use of DMF for oral treatment of relapsing-remitting MS (RRMS) as well as a number of *in vitro* studies on MS-relevant cells have shown very promising results.

## 10. Clinical Studies

Schimrigk et al. [92] performed the first open-label, baseline controlled clinical study with Fumaderm in patients with RRMS. They showed that Fumaderm treatment significantly decreased the number and volume of gadolinium-enhancing lesions. In addition, they reported elevated levels of the cytokine IL-10 and decreased expression of the proinflammatory cytokine IFN-*γ*. Furthermore, they found that apoptosis was increased in lymphocytes [92]. In 2008, Kappos et al. [66] published the results of a multicentre, randomized, double-blind, placebo-controlled phase IIb study testing the efficacy and safety of BG-12 (contains DMF only) in RRMS patients. Patients treated with BG-12 showed a dose-dependent reduction of MS lesions compared to the placebo group and there was a trend of a lower annualized relapse rate. BG-12 was generally well tolerated and showed a favorable safety profile [66]. This study was followed by a placebo-controlled phase III clinical study, confirming that BG-12 treatment resulted in a significant reduction of MS lesions compared to placebo. Furthermore, the study showed that the proportion of patients who had a relapse as well as the annualized relapse rate was reduced and a decrease of the disability progression rate was observed in BG-12-treated MS patients [67, 93].

## 11. Mode of Action of DMF in MS

DMF has been shown to initially reduce cellular reduced glutathione (GSH) in different cell types, including neuronal cells [94] and astrocytes [95], which resulted in the activation of the Nrf2 (nuclear factor erythroid-derived-2- (E2) related factor)/Keap-1 pathway in astrocytes, neuronal cells, and primary central nervous system cells [94–96]. A reduction of GSH and an activation of Nrf2 induced the expression of antioxidant enzymes such as NAD(P)H quinone oxidoreductase- (NQO-) 1, glutamate-cysteine ligase catalytic subunit (GCLC), or HO-1, resulting in reduced oxidative stress, proinflammatory cytokine secretion, and proliferation in these cells [87, 94, 95]. Therefore, the activation of the antioxidant Nrf-2 pathway is regarded to mediate DMF's beneficial effects in MS.

## 12. DMF as a Potential Asthma Therapy

In primary human lung mesenchymal cells, DMF inhibited the activation of the proinflammatory transcription factor NF-*κ*B [26, 28, 35]. NF-*κ*B activity was reported to be increased in the airways of asthmatic patients [97]. In addition, proinflammatory cytokines such as TNF-*α* activated NF-*κ*B in ASMC *in vitro*, which resulted in the secretion of a variety of proinflammatory factors including RANTES, CXCL10, or CCL11 [26, 27, 35]. *In vitro,* inhibition of NF-*κ*B downregulated the release of a range of proinflammatory mediators by ASMC [98]. Furthermore, the inhibition of NF-*κ*B reduced airway inflammation in a mouse model of asthma [99]. 

In resting cells, NF-*κ*B is retained in the cytosol in a complex formed with I*κ*B (inhibitor of NF-*κ*B). Upon stimulation, I*κ*B is degraded, which allows free and activated NF-*κ*B to enter the nucleus where it binds to specific NF-*κ*B-sensitive DNA sequences which are located within the promoter regions of many proinflammatory genes [100]. NF-*κ*B activity is regulated by posttranslational modifications such as phosphorylation, glutathionylation, or modification of histones that wind up NF-*κ*B target genes. Protein glutathionylation is a redox-regulated process, whereby a cysteine-thiol of a protein forms a disulfide bond with the cysteine-thiol of GSH [101]. Interestingly, I*κ*B*α* contains cysteine thiols, which are susceptible to glutathionylation [102].

In human cultured ASMC, DMF inhibited the nuclear entry of NF-*κ*B and the binding of NF-*κ*B to the corresponding DNA sequence [26, 35]. In a subsequent study, we provided evidence that the inhibitory effect of DMF on NF-*κ*B nuclear entry is mediated by glutathionylation of I*κ*B*α*, which inhibited its degradation [35]. Furthermore, DMF reduced NF-*κ*B phosphorylation and altered the chromatin environment by inhibiting MSK-1-induced histone H3 phosphorylation in ASMC and keratinocytes [26, 35, 86]. Consequently, DMF inhibited the secretion of NF-*κ*B-dependent cytokines such as interleukin (IL)-6, GM-CSF, eotaxin, RANTES, and CXCL10 when stimulated with TNF-*α* in ASMC and lung fibroblasts [25, 26, 28, 38]. An interesting study by Van Ly et al. has shown that DMF increased rhinovirus (RV) replication and failed to reduce RV-induced IL-6 and IL-8 by human lung fibroblasts [37]. In another study on HIV-infected monocyte-derived macrophages, DMF reduced HIV replication and neurotoxin release [103]. This suggests a virus-specific effect of DMF leaving it an open question whether DMF may help to control virus-induced asthma exacerbations.

In addition to NF-*κ*B inhibition, DMF downregulated the secretion of PDGF-BB-induced IL-6 by airway smooth muscle cells and lung fibroblasts, which was most likely mediated by DMF's inhibitory effect on AP-1 and CREB in these cells [26, 28]. Besides inhibition of proinflammatory cytokine secretion, DMF was shown to reduce PDGF-BB-stimulated ASMC and lung fibroblast proliferation, suggesting a beneficial effect on airway remodeling in asthma [28, 36].

**Figure 3:**
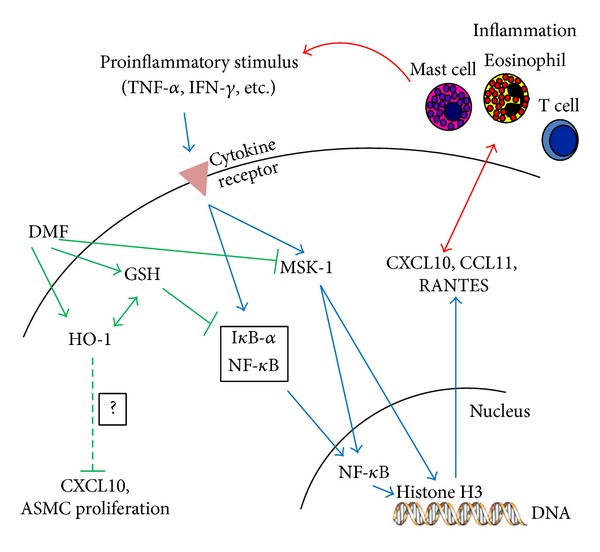
Secretion of chemokines such as CXCL10, CCL11, or RANTES by ASMC attracts immune cells (eosinophils, T cells, or mast cells) into the asthmatic airway. In turn, these immune cells secrete proinflammatory cytokines (TNF-*α*, IFN-*γ*, etc.), which stimulate proinflammatory signaling molecules such as NF-*κ*B or MSK-1 in ASMC, enhancing its proinflammatory function. DMF reduces intracellular reduced glutathione (GSH) level and thereby induces HO-1 expression and I*κ*B-glutathionylation. DMF-induced HO-1 decreased CXCL10 and ASMC proliferation by an unknown mechanism. I*κ*B-glutathionylation inhibited I*κ*B degradation and subsequent NF-*κ*B nuclear entry. Furthermore, DMF inhibited MSK-1-mediated histone H3 and NF-*κ*B phosphorylation, leading to reduced secretion of CXCL10, CCL11, and RANTES. DMF reduces ASMC chemokine secretion and may therefore inhibit the crosstalk between ASMC and immune cells, leading to airway inflammation.

The airways of asthma patients are exposed to increased oxidative stress [104], which may alter ASMC proliferation and proinflammatory cytokine secretion through redox-sensitive signaling pathways [105, 106]. As described earlier, DMF activated the Nrf2 antioxidant response pathway, by reducing cellular GSH levels [94–96]. Once activated, Nrf2 binds to the antioxidant response elements (AREs) within the promoter of antioxidant genes such as HO-1 initiating their transcription. DMF reduced intracellular-reduced GSH level and upregulated HO-1 in ASMC [27, 35]. HO-1 is an inducible enzyme, which protects the lungs from increased oxidative stress [107]. In addition, HO-1 protected the lungs against hyperoxic injury and attenuated allergen-induced airway inflammation and hyperreactivity in animal models of asthma [108, 109]. We demonstrated that DMF induced HO-1 and thereby inhibited PDGF-BB-induced ASMC proliferation and CXCL10 secretion [27, 36]. Importantly, DMF inhibited CXCL10 by ASMCs more efficiently when combined with the GC fluticasone, suggesting a GC sparing effect of DMF [27]. Furthermore, others have shown that activation of Nrf2 and induction of HO-1 inhibit TGF-*β*-induced ASMC proliferation and secretion of IL-6 [110]. In this context, it is of importance that in ASMC-derived from patients with severe asthma the binding of Nrf2 to the antioxidant response elements as well as the expression of HO-1 was reduced when compared to ASMC of nonasthmatic controls [110] suggesting that activation of Nrf2 and upregulation of HO-1 by DMF may compensate this pathology. Together these results emphasize a potential beneficial effect of Nrf2-mediated HO-1 induction in asthma. Regarding other anti-inflammatory actions of DMF, it reduced CXCL10 expression in cells stimulated with either a cytomix (IL-1*β*, TNF-*α*, and IFN-*γ* in combination) or IFN-*γ* alone, which has been shown to be insensitive to GC therapy [25, 27, 41]. Thus DMF may act as an anti-inflammatory drug in steroid-resistant asthma and significantly reduce healthcare costs. A summary of DMF effects on primary lung cells is summarized in Table 1 and the postulated beneficial action of DMF is provided in Figure 3.

## 13. Conclusion

Current asthma therapy is not sufficient to control symptoms in all asthma patients and does not cure the disease. Thus, it is important to find new therapeutic options to treat asthma. CXCL10, CCL11, and RANTES derived from ASMC are believed to be crucially involved in chemotaxis of immune cells into the asthmatic airways and are therefore actively involved in the development of airway inflammation in asthma. DMF reduced the secretion of CXCL10, CCL11, and RANTES as well as ASMC proliferation by inhibiting the proinflammatory transcription factor NF-*κ*B and by upregulation of HO-1. Furthermore, DMF overcame GC resistance and had a GC-sparing effect in a cell culture model of asthma. Taken together, DMF's strong anti-inflammatory and antiproliferative effects in cultured human ASMC suggest that it may be beneficial in asthma therapy.

## Figures and Tables

**Table 1:** DMF effects on primary human lung cells.

Factors	DMF effect	After stimulation	Cell type	Reference
CXCL10	Inhibition at 10–100 *μ*M DMF	TNF-*α* and/or IFN-*γ* and/or IL-1*β*	ASMC	[25, 27]
G-CSF	No effect Inhibition at 10 *μ*M DMF	IFN-*γ* TNF-*α*	ASMC	[27]
Eotaxin	Inhibition at 10–100 *μ*M DMF	TNF-*α*	ASMC	[26, 35]
RANTES	Inhibition at 10–100 *μ*M DMF	TNF-*α*	ASMC	[26, 35]
IL-8	No effect at 0.01–1 *μ*M DMF	Rhinovirus	Lung fibroblasts	[37]
GM-CSF	Inhibition at 100 *μ*M DMF	TNF-*α* and IL-1*β* prior to stimulation with human serum (10%)	ASMC	[38]
IL-6	Inhibition at 10–100 *μ*M DMF	TNF-*α* or PDGF-BB	ASMC and lung fibroblasts	[26, 28]
	No effect at 0.01–1 *μ*M DMF	Rhinovirus	Lung fibroblasts	[37]
Cell proliferation	Inhibition at 10–100 *μ*M DMF	PDGF-BB	ASMC and lung fibroblasts	[28, 36]
